# Considering the role of behaviors in sustainability and climate change education

**DOI:** 10.3389/fpsyg.2024.1394326

**Published:** 2025-04-01

**Authors:** Efrat Eilam

**Affiliations:** Institute of Sustainable Industries and Liveable Cities Program of Arts and Education, Victoria University, Melbourne, VIC, Australia

**Keywords:** climate change education, sustainability education, behavior, collective action, climate action

## Abstract

At the heart of sustainability and climate change education discourse is the notion of student behavioral change, as an emphasized goal. The central positioning of behavior modification raises moral and ethical concerns, as well as concerns regarding the impacts on student well-being. In addressing these issues, this conceptual paper interrogates the role ascribed to student behavior in sustainability education and climate change education. Multiple lenses are applied to critique the behavioral modification approach. Finally, it is proposed to reframe the role of behavior and to conceptualize behavior as forming part of ethics education, where the focus shifts from assigning behavior an instrumental role to conceptualizing its intrinsic educational value.

## Introduction

Notions such as *behavioral change* (Kwauk, 2020), student *activism* (Graham-McLay, 2020) and student *climate action* (Jorgenson et al., 2019), are central to the sustainability education and climate change education discourse (UNESCO, 2017). At the heart of this discourse is the idea that the role of education is to change students’ behaviors. This idea is manifested across extensive sustainability education publications to the extent that UNESCO (2017, 2019a,b) regards behavioral change as a learning dimension on its own right. This central role of behavior is highly unusual in educational discourse, particularly in curricular documents. Examination of literature drawn from Curriculum Theory highlights the a-typicality of changing students’ behavior as a curricular goal. For example: Ross (2000) refers to school curriculum as the broad undertaking in which “anything that schools do that affects pupils’ learning, whether through deliberate planning and organization, unwitting encouragement, or hidden and unrealized assumptions, can all be properly seen as elements of the school’s whole curriculum” (p. 9). Young (2014) proposed a narrower definition suggesting that curriculum is “basically specialized knowledge organized for transmission” (p. 198). In Young’s view curriculum knowledge has two main attributes. First, that it is specialized “in relation to its disciplinary specialist sources” (e.g., science, history), and “in relation to different groups of learners” (e.g., their prior knowledge and year levels) (Young, 2014, p. 198–199). Even when going back to the early 20th century child-centered education, where the focus was on the process of learning, rather than on the contents of learning, philosophers of education, such as Dewey (1902) focused on the learning itself (or rather on what schools do), and not on changing the behavior of the learners as a curricular goal. Furthermore, while curricula often emphasize capacities development, it is quite unusual for any curriculum to claim upfront the need to change student behavior as an educational goal, nor to regard behavior as a central learning dimension in the curriculum. The peculiar role of behavior in sustainability education and climate change education forms the motivation for this conceptual paper. This central positioning of behavior solicits in-depth examination of this educational approach from various lenses, including its educational theory underpinning, and its empirical educational efficacy. Furthermore, the approach raises moral and ethical concerns, as well as concerns regarding the impacts on student well-being. In addressing these issues, this paper aims to interrogate the role ascribed to student behavior in sustainability education and climate change education, by examining and critically discussing a range of sustainability education and climate change education literature.

The paper begins by first discussing the conceptualization of behavioral change as a means and as a goal. This is followed by presenting empirical evidence regarding the efficacy of the approach. It continues to discuss the literary debate regarding individual behavior change *versus* collective action. This is followed by presenting a range of literature criticizing the behavioral change approach, using different lenses for examination. The literature is further interrogated to examine the relationships between behavioral change and other educational outcomes, including knowledge acquisition, attitudes and emotions. Finally, I offer a different lens for reframing the role of behavior in climate change education.

Before progressing further, it is worth noting that the intention in this paper is to contribute primarily to the field of climate change education. However, since the roots of climate change education are so heavily tied up with sustainability education, it is not possible to address the role of behavior in climate change education without discussing the conceptualization of behavior in sustainability education. Regarding the use of terms, in this paper the term *sustainability education* is used interchangeably as a generalist term, encompassing a range of terms used for addressing the broad field, spanning across environmental education, education for sustainable development (ESD), global citizenship education, education for environmental sustainability and others. The term *climate change education* is considered here as separate and distinguishable from *sustainability education*. However, the question of whether climate change education is independent from sustainability education, is contested and unresolved in the literature. While sustainability education literature proclaims to include climate change as a theme of sustainability (UNESCO, 2021a), some researchers suggest addressing climate change education as a field on its own right (Eilam, 2022).

## Behavioral change conceptualization in sustainability education literature

Behavioral change forms a primary goal in sustainability education (Rousell and Cutter-Mackenzie-Knowles, 2020). The idea that the learning outcomes of education should include the changing of students’ behavior or encouraging them to take actions has its roots in the early conceptualization of environmental education. The 1978 Tbilisi Declaration stated as a goal for environmental education to “create new patterns of behavior of individuals…” (UNESCO, 1978). Later in the 1980s following debates as to whether it is ethical for schools to prescribe behavioral goals, particularly when the desired behavioral outcomes are unclear and contested at times, a new educational model was proposed, shifting the educational goals from prescribed behaviors to what was termed Action Competence (Mogensen and Schnack, 2010). This softer approach, while continuing to focus on students’ behavior in relation to their social and physical environment, put more emphasis on the development of capacity to act in the public sphere, rather than on prescribing the desired behaviors. However, Blum et al. (2013) reported that in the United Kingdom both approaches were contested, and it was debated whether schools should be allowed to teach for behavior, as opposed to helping students to deal with arising uncertainties. However, in current sustainability education literature, behavior continues to play a central role, where the most prevailing approach is to conceptualize households and schools as “the primary contexts for action and children and youth the primary agents of change” (Jorgenson et al., 2019, p. 165).

In sustainability education literature, behavioral change plays a dual role. It serves both as means to achieve other sustainability education goals, and as a goal on its own right. As means, the basic idea is that if every person changes their behavior, the world will become sustainable, thus the goal of sustainability may be achieved, through individuals’ responsible behaviors. Additionally, individual behavioral change serves as means for mobilizing societal change. Thus, individual behavior has two main manifestations, at the personal household level, where students need to change their individual daily behavior; and at the societal level, where students are expected to mobilize change in society, what is often referred to as *agents of change* (Jorgenson et al., 2019).

As a goal, the rational for behavioral change is that focusing on students’ everyday behavior enables to empower students, increase a sense of agency, and prevent a sense of despair and helplessness (Jorgenson et al., 2019). According to this perception, climate change education needs to focus on “‘local, tangible and actionable’ aspects of climate change that can be ‘addressed by individual behavior’ (Anderson, 2012, p. 197)” (Jorgenson et al., 2019, p. 165).

These conceptualizations of the roles of behavior, as a goal and as a means, appear repeatedly in sustainability education literature. For example, UNESCO (2017) suggests that “to create a more sustainable world …individuals must become sustainability change-makers” (UNESCO, 2017, p. 7). This idea is expanded upon as follows:

ESD aims at developing competencies that empower individuals to reflect on their own actions, taking into account their current and future social, cultural, economic and environmental impacts, from a local and a global perspective. Individuals should also be empowered to act in complex situations in a sustainable manner, which may require them to strike out in new directions; and to participate in socio-political processes, moving their societies toward sustainable development (UNESCO, 2017, p. 7).

According to UNESCO (2019a), Target 4.7 in SDG 4 Quality Education, aims to “empower learners to assume active, responsible and effective roles to tackle challenges at local, national and global levels” (UNESCO, 2019a, p. 2). These ideas connecting individual behaviors to large scale changes in society are further explicated in UNESCO, 2020 Roadmap (UNESCO, 2020), where it delineates the role of education as a means “to bring about the fundamental behavioral shift to sustainable development” (p. 9).

The idea of big transformation implies changes in individual action intertwined with reorganization of societal structures, and it requires ESD to track the transformation… Fundamental changes required for a sustainable future start with individuals. ESD has to place emphasis on how each learner undertakes transformative actions for sustainability (UNESCO, 2020, p. 18).

According to this perception, education has a clear role of transforming individual behavior, and the achievement of this goal needs to be monitored on an individual level. Thus, educational assessment is required to track each student’s behavior, and measure the individual achievement of this educational outcome. This view concerning the role of education is referred to in the literature as *Individuation* approach (Olsson, 2021). In the context of this report the term *individuation messaging* is used to describe an educational approach that conveys the message to students that they bear personal responsibility to solving the climate change problem through their individual daily behavior.

The *individuation messaging* is at the heart of the ESD agenda. In its essence it represents a positivist, reductionist view that the whole is a linear sum of its parts, and that if everyone behaves sustainably, the world will become sustainable, and the problem is solved. These ideas are theoretically and empirically unsubstantiated.

## *Individuation* and the psychological reactance theory

The Psychological Reactance Theory (PRT) (Brehm, 1966) suggests that “when something threatens or eliminates people’s freedom of behavior, they experience psychological reactance, a motivational state that drives freedom restoration” (Rosenberg and Siegel, 2016, p. 1). According to Brehm’s theory, when people encounter restrictions to behaviors, which they have previously exercised freely, they will be motivated to restore their freedom of behaviors. This theory provides the theoretical basis for predicting that individuation messaging aiming to restrict students’ range of behaviors to a more limited set of permitted behaviors, is likely to be met with resistance.

## Empirical findings concerning the efficacy of cultivating behavioral change at schools

Studies examining the short- and long-term effects of sustainability education programs on students, reveal that the efforts to change students’ behaviors were often unsuccessful. A study examining 38 eco-schools in Flanders, compared to 21 control schools, revealed that in the eco-schools, students’ knowledge increased. However, there was no effect on their behavior (Boeve-de Pauw and Van Petegem, 2013). Similarly, large-scale research on the sustainable-schools certification in Canada revealed no effect on students’ environmental behavior (Niebert, 2019). A longitudinal study reveals that students develop pro-environmental behaviors at ages 7–10, and that this effect drops at ages 14–18, regardless of increases in scientifically accurate knowledge (Otto et al., 2019). This suggests that even when educational efforts are successful in increasing intention to act, these effects wear off as children grow. This wearing off may potentially be attributed to students’ increased understanding concerning environmental issues, a relationship discussed further below.

One way for explaining the consistent lack of success in promoting individual behavior, is by drawing upon Weckroth and Ala-Mantila (2022) discussion regarding socio-spatial boundaries in determining behavior. This perspective suggests that individual behaviors are never performed in an isolated manner, and they are always socio-spatially bounded. People naturally adopt to the socioecological systems in which they live, and these systems in turn pose constraints on behavior. It follows, that when considering students’ behavior, there is a need to consider the socio-spatial contexts that operate beyond the schools and their influences. This means that when students live in an environment which is essentially consumerist in its behavior, it inevitably limits the opportunities for students to behave in non-consumerist ways, to the extent that such environments convey the message to students that non-consumerist behaviors are meaningless within their contexts. Thus, once again pointing to the importance of making changes at the system level, rather than at the individual level.

## *Individuation* versus collective action

The literature differentiates between *individual behavior* and *collective action*. *Individual behavior* includes the range of behaviors that people can do in their private sphere, such as walk or cycle to work, rather than drive a car, or reduce households’ consumption. *Collective action* refers primarily to participation in social movements related to climate change, such as climate strikes (Jorgenson et al., 2019).

Unlike the individual behavior, where people perform certain behaviors mostly related to reducing their consumption, or what is known as *carbon footprint*, in *collective actions* people come together to express their views and values and exert influence on decision makers that have the power to make changes at the system level. Thus, while *individual behavior* relates to the realm of behavior acquisition, *collective action* relates to the realm of *attitude acquisition*. In educational settings, teaching for *behavioral change*, puts forward the expectation that students change their behavior, whereas teaching for *collective action* puts forward the expectation that students express their attitudes and opinions in the public sphere. The literature does not seem to make this conceptual distinction and refers to both as *behavior*. However, the literature does question the value of cultivating *individual behavioral change* as compared to cultivating *collective action*, where both are essentially perceived as different forms of behavior (Reimers, 2021).

Jorgenson et al. (2019) criticizes the *individuation* approach and perceive it as a residue from the early EE approach in the 1970s. Their review examined the expressions of this approach in educational interventions. Table 1, adopted from Jorgenson et al. (2019) clearly reveals that most educational programs focus on behavioral change at the private sphere.

**Table 1:** Recent EE research that uses the energy behavior of individual persons to measure the effectiveness of educational interventions.

Primary focus	Population	Energy behaviors measured	Type
Climate change education	Secondary school students	Energy conservation and consumption	Private sphere
Climate change education	Young adults	Energy conservation	Private sphere
Energy education	Elementary school students	Energy conservation	Private sphere
Climate change education	Adults	Energy conservation and consumption	Private sphere
Environmental education	Small business owners	Energy conservation and consumption	Private sphere
Environmental education	University students	Energy conservation	Private sphere
Environmental education	University students	Energy conservation	Private sphere
Environmental education	Adults	Support for renewable energy subsidies	Policy support (individual)
Energy education	University students	Persuading people to conserve energy	Non-activist political (individual)
Energy education	Elementary school students	Energy conservation and consumption	Private sphere
Climate change education	Secondary school students	Support for adaptation and mitigation efforts Energy conservation	Policy support (individual) Private sphere

Adapted from Jorgenson et al. (2019).

Jorgenson et al. (2019) note that in various studies *collective action*, rather than being conceived as a social collective action in pursuing shared interests, it is conceptualized as the sum of individual actions, such as the summation of the number of households that reduced their electricity consumption. Reimers (2021) criticized the educational focus on influencing individual behavior, claiming that in effect this is a form of privatizing climate action, and

reinforcing a simplistic and narrow conception of the relationship between climate change, human action, and energy system change and distorting the fact that many of the most impactful climate actions are decisions about energy supply systems that are made by state and market sector actors under direct pressure from advocacy coalitions and other social collectives (Reimers, 2021, p. 19).

Similarly, Kranz et al. (2022) stress that “greater effectiveness has been attributed to actions in the public sphere than to the actions of individuals” (p. 1), where people are exerting pressure on governments to make system changes. However, they observe that in sustainability education, “the responsibility for the emissions is often attributed to large-scale societal actions, while mitigation actions focus on private and technical/scientific strategies and voluntary agreements” (Kranz et al., 2022, p. 20). This approach of delegating the responsibility for mitigation and adaptation to individuals received a range of criticism, related to supporting neo-liberalism, its strategic ineffectiveness, its underlying social engineering, and the negative emotional impact on students. These criticisms will be discussed later in this paper.

## Criticizing the behavioral change approach

### *Individuation* supports neo-liberalism

According to the neo-liberal view individuals are autonomous, free to choose a course of action, and thus assumed to be the primary agents of social change through their individual choices. It follows that the unsustainable state of the planet can be attributed to individual choices. Thus, the failure of students to make the correct choices regardless of the efforts of the education systems to encourage them do so, suggests that this must be each student’s individual failure (Olsson, 2021).

This neo-liberal worldview was criticized for serving the capitalist free market, as it privatizes the need for climate action. By delegating the responsibility to individuals, educators may be inadvertently drawing attention away from where the problem essentially relies, thus enabling governments and industries to continue business as usual (Kenis and Mathijs, 2012; Ojala, 2015).

According to Kwauk (2020) the sustainability education agenda was “co-opted by neoliberal proclivities: Individual action and behavioral change prioritized over collective action and structural change” (p. 10).

As a result, education systems around the world continued to focus on preparing children, youth, and adults “to join the local labour market to nourish the global marketplace and satisfy corporate needs” (Jickling and Wals, 2008, p. 2)—now under the guise of achieving sustainable development (Kwauk, 2020, p. 10).

Thus, the disproportionate responsibility that is placed on individuals may be regarded as a neoliberal tactic to evade governments and corporates’ responsibilities, by diverting the problem to the *down- stream* symptoms rather than the *upstream* causes (Bellino and Adams, 2017; Uzzell and Räthzel, 2009).

### The strategic ineffectiveness of *individuation*

Individual behavioral change is relatively insignificant in impacting climate change. When 100 companies across the globe are responsible for 71% of global carbon emissions, what are the chances of any individual action to make a difference on climate change matters? (McManus, 2022). Furthermore, the causes for climate change are rooted in the economic and governance systems, which allow companies almost unlimited use of common natural resources (Niebert, 2019). This begs questioning: If individual behavior is not responsible for causing climate change, how can it be expected to solve climate change? Wouldn’t it be more practical to address the root causes, rather than to expect the recipients of the problem to resolve it? An MIT class estimated the carbon emissions of Americans living in vastly diverse lifestyles, “from the homeless to multimillionaires, from Buddhist monks to soccer moms” (Massachusetts Institute of Technology, 2008, n.d.). The findings were clear. The lifestyle made no difference, they all produced more than twice as much greenhouse gas as the global average. They all lived beyond Earth carrying capacity for atmospheric carbon load (Massachusetts Institute of Technology, 2008). This is because the systems they relied on for sustenance continue to discharge carbon disregarding the differences in individual consumption and lifestyles. These findings clearly suggest that the problem is at the system level, and not a linear sum of the individual consumption patterns.

Viewed from a system level perspective, climate change is a typical case of the Tragedy of the Commons proposed by Hardin (1968). The idea suggests that common resources such as air, water and soil are destined for depletion in the absence of regulation and enforcement. Thus far this idea continues to hold true for shared resources, where there are no direct interactions between the people sharing them, and there is no close physical proximity to the resources. Such is the case concerning resources affecting the climate. This hard-core fact was exemplified in Steinebach (2022) study. The study examined the air pollutant emissions of 14 OECD countries over a period of 25 years (1990–2014). The findings revealed that “only command-and-control (C&C) regulations that are put into practice through well-equipped and -designed implementation structures can be associated systematically with reductions in air pollutant emissions” (Steinebach, 2022, p. 255). All other approaches trialed, including softer approaches aiming to stimulate more environmentally friendly behavior by “assisting business and individuals by providing information on environmental issues” (p. 227), had no effect whatsoever.

The history of major social changes such as illegalizing slavery and women’s suffrage provide further evidence for the ineffectiveness of individual behavioral change. For example, slavery was not abolished because individual slave owners released their slaves. It was abolished because philosophers and public influencers in the west have questioned the moral basis of the practice. Two centuries of public debate have led to Britain being the first Western country to pass the Slave Trade Act in 1807, making it illegal to engage in the transatlantic slave trade (Oldfield, 2013). Similarly, the global struggle for gender equality, and ultimately its expression in legislative acts, such as women’s right to vote, did not come about due to individual women exerting equal rights and responsibilities to those of their husbands in their individual households. It came about through centuries of philosophical scholarship accompanied by social movements calling for change (Offen, 2000).

History reveals that big changes were led by mounting public pressures on governments to change legislation. Thus, it was public expression of attitudes that drove the changes, not individual behavior (Eilam and Trop, 2012; Niebert, 2019). Niebert (2019) explicates that it is not the individual abandonment of CFC-containing deodorants, not the individual change of your electricity provider from nuclear to green energy and not our individual decision to buy an electric car instead of a fossil car, that drives the world into a green state. It is hard political and economic decisions that make a difference (p. 3).

### Corporates’ engagement in social engineering

The apparent ineffectiveness of individual behaviors in impacting system change, begs the question of: What might have influenced education systems to becoming so preoccupied with individual *carbon footprint*? One possible explanation may have to do with purposefully designed *social engineering*. Here *social engineering* is defined as “any act that influences a person to take an action that may or may not be in their best interests” (Security Through Education, 2024). Various publications point to a concerted effort since the late 1980’s, by polluting corporates to purposefully engage in social engineering by shifting public attention from the corporates’ responsibilities to individual responsibility. One example is the establishment of the Global Climate Coalition (GCC) in 1989 in response to the establishment of the IPCC in 1988, in UN Resolution 42/187 (United Nations General Assembly, 1987). The GCC was a consortium comprised of over 40 of the biggest and most polluting corporates in the United States. Equipped with a total estimated expenditure of $8.3 million, the GCC’s sole purpose was to manipulate the IPCC and undermine the climate change science. A review of the activities of this coalition revealed that.

the GCC engaged in four distinct activities to obstruct climate action: (1) monitoring and contesting climate science, (2) commissioning and utilizing economic studies to amplify and legitimate their arguments, (3) shifting the cultural understanding of climate change through public relations campaigns, and (4) conducting aggressive lobbying of political elites. Through these activities, the GCC played an important role in obstructing climate action, both in the U.S. and internationally (Brulle, 2022, p. 1).

GCC was not alone in the corporate world, and soon after its establishment it became standard practice for the fossil fuel industry and other polluting industries to sponsor astroturfing organizations with misleading euphemistic names such as “National Wetlands Coalition,” “Greening Earth Society,” “Washington Consumers for Sound Fuel Policy” or “American Coalition for Clean Coal Energy” (Grolleau et al., 2022). Under the guise of grassroot environmental protection, these organizations were well positioned to convey misleading educational campaigns. Consequently, over the past four decades a sophisticated machinery of marketing companies and lobbyists, were established with the purpose of using whatever means available to create the social-political conditions that would allow them to continue *business as usual*. These strategies included among others, media campaigns for convincing the public that the responsibility for solving the problem lays in their hands, and if they change their individual lifestyle, the problems will be solved. In other words, rather than the corporates being accountable for their role in causing climate change, it is the individual consumer that needs to be blamed and shamed.

According to a blog published in The University of Melbourne Scientific Scribles (2021), one story describing the propagation of the *individuation* approach, goes as follows: In the early 2000 the oil company British Petroleum (BP) hired the public relations company Ogilvy & Mather to manage their public image. This company came up with the idea of diverting public attention from the company’s emissions [estimated at 340 million tons CO_2_ equivalents per year in 2020 (GlobalData, 2024)] to individual households, by promoting the concept of *carbon footprint*, and the idea that individual households are responsible for the carbon emissions. By 2004, 278,000 users were already calculating their *carbon footprints*, and soon after, whole school programs were planned around *carbon footprint* calculations.

Consequently, while researchers in the field of climate change emphasize change at the political, economic and governance levels, sustainability educators and researchers continue to promote *individuation* regardless of its ineffectiveness (Jorgenson et al., 2019; Waldron et al., 2019). The stronghold of the *individuation* approach among sustainability educators may be viewed as a testimony for the success of the various social engineering campaigns. This can be exemplified in a European Commission report claiming that the aim of sustainability education is to “empower individuals to reflect on their own actions, taking into account their current and future social, cultural, economic and environmental impacts from both a local and a global perspective” (Mulvik et al., 2022, p. 13). Even more concerning is that ESD not only cultivates the unsubstantiated idea that individuals can change the course of climate change, but also participates in assisting corporates and governments in cultivation the faulty message that while the economic-governance model has created the problem, it is the education system that can solve the problem. This idea is expressed as follows: “Formal education can play a particularly strong role in mitigating climate change, as well as responding to its impact” (Mulvik et al., 2022, p. 10). These ideas put students at risk of developing adverse mental health, as discussed in what follows.

### The emotional impact of *individuation*

The *individuation* approach was criticized for repackaging the early 20th century Behaviorism and bringing it back into schools. Once again, we are seeing an educational approach which objectifies students through conditioning methods of rewards and punishment of behavior, where students are praised for changing behavior and let to feel guilty if they do not. Critiques of *individuation* claim that “in such approaches, people are considered as objects to be conditioned rather than that they are taken seriously as subjects of change” (Kenis and Mathijs, 2012, p. 53).

Kenis and Mathijs (2012) addressed the negative psychological effects of the *individuation* approach. They noted a sense of stress that may be interpreted as an outcome of guilt feelings that arise around the pressure to perform *responsible environmental behaviors*, as follows:

One respondent sent us two text messages after the interview, because she remembered a few of her individual actions that she forgot to mention during the interview. Only when we clearly stated that they could interpret engagement in a broad way, from reading about the environmental issue to signing petitions and so on, the respondents seemed to feel relieved, stopped focussing on their own individual behaviour change, and even started to severely criticise this strategy (Kenis and Mathijs, 2012, p. 55).

Hogg et al. (2021) developed and trailed a scale for measuring eco-anxiety. Their research revealed an important distinction between two types of anxiety. The first is anxiety directly related to the state of the environment, and the second is anxiety derived from one’s concerns regarding their own impact on the environment. This finding has an important implication for education. It suggests that educational approaches that promote the message of individual responsibility, are increasing the likelihood that students will develop anxiety concerning this issue. Furthermore, Hogg et al. (2021) note that “rumination and personal impact concerns may persist to a greater extent over time as they are driven and maintained by cognitions (e.g., thoughts about the environment and one’s personal behaviors)” (p. 7). This suggests that inducing students to change their behavior as means for solving climate change, has both short term and long-term effect of causing anxiety.

### How does increased climate change knowledge relate to behavioral and attitudinal-emotional changes?

The sustainability education literature suggests that increased knowledge concerning climate change may be associated with increased intention to change behavior (UNESCO, 2021a,b). It is also assumed that by promoting individual behavioral change in everyday context, students will be empowered to become change agents, “increase their understanding and engagement, and avoid the despondency and helplessness that climate change can foster” (Jorgenson et al., 2019, p. 165). The evidence does not support these assertions, in fact it points to the opposite. In what follows, the relationships between increased knowledge and behavior are discussed, followed by examination of the relationships between increased knowledge and other emotional and attitudinal aptitudes.

### The relationship between knowledge and individual behavior

Research examining the relationships between increased climate change knowledge and increased performance of pro-environmental behaviors reveal that the correlations range between negative to weak correlations (Busch et al., 2019; Kranz et al., 2022). The evidence for these relationships come from multiple studies examining multiple aspects of the relationships between climate change knowledge and individual behavior.

A study by Kenis and Mathijs (2012) among 12 environmental activists, found that common to all of them was a sense of powerlessness in the face of climate change, and lack of belief in individual action, as means for addressing climate change. These environmentally informed people stated that “they used to be very strict on their individual behavior in the past but became less rigid in this because of their doubts about the usefulness of this type of action” (p. 52). This suggests that people who are at the frontline of working on climate change issues have less faith in the usefulness of individual actions to impact the course of climate change.

Similarly, Organisation for Economic Co-operation and Development (2018) Programme for International Student Assessment (PISA) showed that while 79% of 15-year-olds students knew about climate change, only 57% of the students felt that they could do something about climate change (Organisation for Economic Co-operation and Development, 2018). Similar to the above findings among adult environmental activists, the PISA results showed that among youth, increased knowledge about climate change is associated with a sense of powerlessness, and less faith in the power of individual behaviors to make a difference (European Commission, 2022; Schleicher, 2021).

Powdthavee (2020) examined the relationships between raising of the minimum school leaving age from 15 to 16 years of age, and the acquisition of pro-environmental behavior among 20,000 England-born citizens. The findings revealed that increased level of understanding of the causes of climate change did not result in increased intention to behave in pro-environmental behaviors. Furthermore, more education was correlated with more belief that the environmental crisis is beyond repair. Powdthavee (2020) concluded that “although more education had managed to have a desirable impact on the participants’ understanding about the causes of climate change, it did not effectively increase their willingness to change their behaviors to help save the environment” (p.13).

A UNESCO (2021b) study among teachers found that while 40% of teachers reported confidence in teaching climate change knowledge, only 20% were able to explain how to reduce their carbon footprint. These findings suggest that there is a rhetoric-practice gap between what UNESCO’s publications idealize and what the teachers themselves are doing. Additionally, the findings once again point to the low association between climate change knowledge and individual behavioral change. Similar results were obtained in a study examining the impact of an educational intervention among 628 Australian adults. The intervention consisted of increasing the participants’ knowledge concerning the negative impacts of the palm oil industry on the environment, and the importance of purchasing sustainable palm oil, as well as providing information regarding various behaviors that individuals can perform to help promote the use of sustainable palm oil (Sundaraja et al., 2022). The findings revealed that while the participants’ knowledge and awareness about the issue significantly increased, this had no effect on the participants’ consumer behavior, and could have even had potentially negative effect. Contrarily, the control group who received no knowledge and training concerning sustainable palm oil, showed more pro-environmental consumer behavior in relation to palm oil. The authors suggested that the increased understanding of the complexity of the issue, may have acted to inhibit pro-environmental consumerism (Sundaraja et al., 2022).

Finally, some reports suggest that people involved in environmental activism, tend to have lower scientific knowledge about the issues (Rousell and Cutter-Mackenzie-Knowles, 2020). This was demonstrated in Kranz et al. (2022) study that found negative correlation between environmental understanding and performance of pro-environmental behaviors. Participants who had higher environmental understanding had a higher carbon footprint than those who were less aware. Furthermore, the study revealed that the best predictor of low consumption is people’s income, not their environmental awareness.

One possible explanation for the findings that people who understand more act less, and people who understand less act more, is that the growing understanding of the climate change problem brings about more accurate appraisal of the situation, and a more realistic assessment of people’s individual abilities to make a difference. This leads to the rational conclusion that individual behavior will not make a difference in the big scheme of climate change. Thus, the findings once again demonstrate that increasing climate change knowledge while advocating for individual reduction of resource consumption, as means for solving climate change, is ineffective and counterproductive.

## The relationships between climate change knowledge and attitudinal-emotional aptitudes

As discussed above, the *individuation* approach has direct negative effects on people’s state of mind. However increased climate change knowledge seems to also play a role in impacting states of mind, in interaction with *individuation*.

Clayton (2020) proposed a psychological explanation for the interactions between the three factors: increased climate change knowledge, exertion of pressure on students to solve the problem through individual behavior, and a sense of helplessness. Her explanation suggests a psychological coping mechanism by which when students appraise the problem as not being amenable to solution, yet at the same time they are encouraged to solve the problem through individual behavior, this may lead to distress, which in turn may lead to a range of responses, including pessimism, depression, anxiety or apathy.

At the other end of the spectrum is the lack of climate change knowledge. Less climate change knowledge seems to be associated with skepticism, climate change denial, and naïve optimism. In skepticism and denial there is disbelief in the extent of the problem (Busch et al., 2019; Stevenson et al., 2020). In naïve optimism, there is an assumption that the problem is solvable, and that it is likely to be solved (Schleicher, 2021). The literature associate skepticism and denial with low engagement with environmental behaviors, whereas naïve optimism is associated with positive engagement in environmental behavior (Ojala, 2013).

The notion of *hope* was also addressed in the literature in relation to behavior. Armstrong and Krasny (2020) suggest that engagement with pro-environmental behaviors is associated with hopefulness about combating climate change. Ojala (2013) offered the notion of *constructive hope* to signify effective coping mechanisms versus ineffective. Ojala (2012, 2016) identified a coping mechanism, which she termed *meaning-focused* strategy for coping with the emotional effects of climate change. This notion corresponds with the work of Frankl (1988) and is closely related to developing *hope* for a better future. In Ojala (2018) body of research, this form of coping essentially involves reappraisal of the situation to make it seem less stressful, leading to a positive engagement with climate change. While this finding suggests a form of *hope* that may be associated with engagement in environmental behavior, it may also be viewed as another form of naïve optimism. The reason is that *meaning-making* as a form of coping by *hope*, is not a stand-alone construct. It needs to be evaluated against the prospect of resolution of the problem. While Frankl’s notion of *meaning-making* was constructed as a coping mechanism for survival with a realistic hope of resolution, climate change is a different case altogether. In the case of climate change, there is a scientific consensus that climate change will worsen, not get better. Viewed from this perspective, hope may be regarded as equivalent to naïve optimism and thus may be associated with low climate change knowledge (concerning the gravity of the situation and its unlikely solvability), and increased willingness to perform environmental behavior.

Overall, there seems to be strong indication that encouraging students to perform individual behavioral changes for the purpose of solving climate change is misleading, ineffective and psychologically damaging.

## Conclusion: reframing the role of behavior in climate change education

If the role of behavior is not to solve the climate change problem, then does behavior have any role at all to play in climate change education? Another way of asking this question, is as follows: Why teach behaviors such as refraining from using disposable products or walking to school instead of being driven, if it makes no difference whatsoever in relation to the state of climate change?

The proposed answer to this question is: Because these are the right thing to do. These norms of behavior reflect the values that we as society wish to instill in our children. Stemming from a universal ethics perspective, our role as educators is to teach our students the set of values and ethical behaviors that need to regulate and underlie the relationships between humans and Earth.

The reason for teaching environmental behavior at schools should not be different than the reasons for teaching students not to bully each other at the playground. We teach not to bully, not because we wish students to go out into the world and solve countries’ territorial conflicts with each other, such as governments’ bullying behaviors toward neighboring counties. We do so, because we wish to educate humans that are capable of respectful and ethical conduct among each other, and in their communities. Similarly, we teach students not to bully the Earth by unnecessary consumption, not because we wish them to solve climate change, they cannot, and it is inappropriate to expect them to do so. We teach these norms of behavior because we wish to raise human beings who are respectful of the Earth and express their appreciation for Earth’s limited resources by not trashing it, and through other forms of respectful behavior.

Viewed from this perspective, behavior plays a critical role in ethics education. It is the normative-behavioral expression of the values and ethics that need to govern societies’ conduct and students included. It is not a means for solving the climate change problem. The climate change problem was not created by the education system, and it will not be solved by the education system. Yet regardless, education has a critical role in preparing students for living in climate change and in cultivating the ethics and norms of behaviors that need to guide them through life.

The difference between the two views concerning the role of behavior is fundamental, where according to one view behavior plays an instrumental role and according to the other view it forms an educational end goal. The instrumental view of education, by which behavioral acquisition serves ulterior purposes reflects a neo-liberal worldview. According to this view, all things are judged by their instrumental value. Ethics is perceived as relativist, pluralist, unbounded by ethical universalism. Thus, students are led to judge the worth of their individual behaviors by the extent to which their behavior helped solve the climate change problem. However, when behavior is framed within ethics education, the education itself becomes the goal. Here the focus shifts from solving the climate change problem to focusing on educating the students and preparing them to living in a climate change era. This perception aligns well with Biesta’s conceptualization of the three schooling domains of purposes, which include: qualification, socialization, and subjectification. Where qualification refers to schools’ role in transmission of knowledge and skills. Socialization refers to the representation of cultures traditions and practices, including cultural norms and values. And subjectification refers to the growth of students as individuals, the opportunities and restrictions provided to them to realize their potentials. Essentially it relates to “how I exist as the subject of my own life, not as the object of what other people want from me” (Biesta, 2020, p. 93). The elimination of *individuation* in sustainability education and climate change education enables to refocus education on these three domains of purposes, where behavior plays a role in educating the students across the three domains.

The dissociation of behavior from its instrumental goal, eliminates many of the negative impacts of *individuation*, outlined above. For example, students will be freed of guilt feelings and anxiousness associated with their behavioral impact on climate change. Furthermore, once behaviors are dissociated from saving the planet, students may be more inclined to perform environmental behaviors, as they do so because it is the right thing to do, not as means to solve climate change. It also follows that the association between increased climate change knowledge and decreased behavior will no longer prevail. This is because when behavior is framed as a normative ethical act, it is not aimed to solve climate change in the first place, thus the intrinsic value of the behavior continues to hold, irrespective of increased knowledge regarding the uselessness of the behavior in solving the problem. This was expressed well by environmental activists in Kenis and Mathijs’s (2012) study, where “almost none of the respondents said they believe that individual behavior change could make a real contribution to tackle climate change. The arguments given for this kind of engagement were all of an ethical nature, they were about ‘doing the right thing’” (p. 51). Indeed, environmental behavior is no more and no less than doing the right thing. Furthermore, Niebert (2019) suggests that if we relieve teachers from the need to promote individual behavioral change that does not work anyways, they will be free to focus on providing in-depth climate change education that addresses the real underlying systemic issues.

To conclude, behavior has and always had an important role to play in educating young people. However, in recent years it appears that the sustainability education agenda has hijacked behavior and reframed its role in the service of ulterior purposes. The present paper presented strong evidence for the ineffectiveness of this approach, its moral lacking, and its potential harm to student well-being. By reinstating the role of behavior as intrinsically valuable within the framework of ethics education, behavior could once again play its valuable role in the complex undertaking of educating young citizens to live as well as they can within their societies.
